# Astaxanthin supplementation enriches productive performance, physiological and immunological responses in laying hens

**DOI:** 10.5713/ab.20.0550

**Published:** 2020-12-01

**Authors:** Yuanzhao Zhu, Long Yin, Jinshan Ge, Xuezhuang Wu, Yuhan Peng, Tao Zhang, Meihong Jiang

**Affiliations:** 1 Key Laboratory for Animal Nutritional Regulation and Health of the Anhui Province, College of Animal Science, Anhui Science and Technology University, Bengbu 233100, China; 2 Shandong Zhongcheng feed Technology Co., Ltd, Feicheng, 271600, China

**Keywords:** Astaxanthin, Laying Hens, Egg Quality, Antioxidant Defenses, Immune Defenses

## Abstract

**Objective:**

Astaxanthin is a natural super antioxidant. The present study was carried out to investigate the effect of astaxanthin rich *Phaffia rhodozyma* (PR) supplementation in diets on laying production performance, egg quality, antioxidant defenses and immune defenses in laying hens.

**Methods:**

A total of five hundred and twelve 60-week-old Lohmann Brown laying hens (2,243±12 g) were randomly assigned to four groups, each including 4 replicates with 32 birds per replicate. Astaxanthin rich PR was added to corn-soybean meal diets to produce experimental diets containing 0 (Control), 800 mg/kg, 1,200 mg/kg, and 1,600 mg/kg PR, respectively. The astaxanthin content in the diet was 0.96 mg/kg, 1.44 mg/kg and 1.92 mg/kg respectively.

**Results:**

Results showed that dietary PR supplementation tended to increase daily feed intake (p = 0.0512). There was no effect of astaxanthin rich PR on Haugh units, albumen height, egg shape index, eggshell strength, and eggshell thickness at weeks 6 (p>0.05). However, egg yolk color was significantly improved (p<0.05). In addition, astaxanthin rich PR supplementation significantly increased serum glutathione peroxidase and superoxide dismutase activity (p<0.05), increased serum immunoglobulin G content (p<0.05), and reduced malondialdehyde content (p<0.05) in laying hens.

**Conclusion:**

In conclusion, astaxanthin rich PR can improve the color of egg yolk, enhance the antioxidant defenses, and regulate the immune function.

## INTRODUCTION

Astaxanthin, also known as allophycocyanin, is found in various microorganisms and marine animals, such as *Phaffia rhodozyma* (PR), microalgae, salmon, krill, complex plants and some birds [1]. Natural astaxanthin is mainly extracted from seafood [2]. Astaxanthin was first extracted and identified from lobster by German Chemist Richard Kuhn in 1938. The antioxidant activity of astaxanthin is much better than other carotenoids, and its ability to scavenge singlet oxygen is 6,000 times higher than that of vitamin C [3]. According to Shimidzu et al [4], the antioxidant capacity of astaxanthin in deuterated trichloromethane is 540 times higher than that of vitamin E in a mixed solution of deuterated trichloromethane and deuterated methanol, astaxanthin’s ability to scavenge singlet oxygen free radicals is 40 times higher than that of carotene. It is well know that astaxanthin has an important role in the prevention of atherosclerosis [5]. With the prohibition of antibiotics, astaxanthin has become a potential green additive because of its natural, non residual, antioxidant and immune function. However, there are few studies on astaxanthin application in animal production.

Previous studies have shown that dietary astaxanthin supplementation can improve the egg quality of hens [6] and ducks [7]. Consumers are interested in eggshell color. The color of eggshell is mainly affected by the environment and physiological conditions of the laying hens [8]. The eggshell color comes from vitellogenin, the precursor of which is carotenoid [9]. In addition, the addition of astaxanthin delayed the decrease of yolk index and yolk color [10]. Therefore, it is speculated that astaxanthin can prolong the storage time of eggs. For laying hens and Pekin ducks, astaxanthin enhanced the antioxidant capacity [7] and regulated lipid metabolism of laying hens [11]. For broilers, the addition of astaxanthin could significantly improve muscle color [12] and alleviate structural damage to the digestive system [13]. Meanwhile, the feed industry has been advised to add functional ingredients to conform with consumers’ preferences. So astaxanthin has become a popular research topic.

There are two sources of astaxanthin, artificial synthesis and biological acquisition [14]. The synthetic astaxanthin is cis structure, and its bioavailability is very low [15]. The natural astaxanthin is trans structure, which has biological activity and is relatively stable. The PR is an ideal source of astaxanthin. At present, consumers are genuinely concerned about the use of additives in food, and their interest in natural substitutes is also increasing. However, there are few reports on the study of natural astaxanthin from faff yeast in laying hens.

Therefore, the purpose of this experiment was to determine the effects of dietary astaxanthin rich PR on laying performance, egg quality, antioxidant defenses and immune defenses and to evaluate the value of astaxanthin in improving yolk color.

## MATERIALS AND METHODS

All procedures of this experiment were approved (ethical committee number: 202005) by the Animal Ethics Committee of Anhui Science and Technology University (Fengyang, China).

### Birds, diets, and experimental design

Five hundred and twelve 60-week-old Lohmann Brown laying hens (2,243±12 g) were randomly divided into 4 groups. Each group had 6 replicates of 15 hens each. The birds were kept in a three-tier battery cage, each cage (width 400 mm, length 450 mm, height 450 mm) including 4 birds. The experiment lasted for 6 weeks, with 16 hours of light and 8 hours of darkness every day. All birds were free to feed and drink. The room temperature was maintained at 18°C to 26°C and the relative humidity was between 55% and 65% via the wet curtain ventilation system.

The experimental diets were formulated according NRC [16] recommendations for laying hens. PR was added to corn-soybean meal diets to produce experimental diets containing 0 (control), 800 mg/kg, 1,200 mg/kg, and 1,600 mg/kg PR, respectively. The astaxanthin content in the diet was 0.96 mg/kg, 1.44 mg/kg, and 1.92 mg/kg respectively. PR was provided by Xi×an Zebang Biotechnology Co., Ltd (Shanxi, China), and the astaxanthin content of PR was 1.2 mg/kg. The experimental diets had the similar nutrient levels and are shown in Table 1.

### Data collection and sample preparation

During the experiment period, feed intake was measured once a week. Eggs were recorded daily in each cage, including egg weight, eggs number. After the experiment, the egg production, average egg weight, daily feed intake and feed conversion ratio (total feed intake/total egg mass) were calculated for the whole experimental phase. At the end of the trial, 12 eggs per treatment (3 eggs/replicate) were collected and weighed. The eggs were stored at room (temperature 25°C±2°C, relative humidity 55%±5%) for 6 days and then were measured for Haugh units (HU), eggshell strength, eggshell thickness, albumen height, egg shape index, and egg yolk color. In addition, eight birds (two birds per replicate) from treatments were selected for blood collection from the wing vein on the 21st and 42nd days. Blood samples were collected and centrifuged at 3,000×g for 15 min. Serum was separated and stored at −80°C for further analyses.

### Sample analyses

At the end of the trial, the egg samples were weighed, and then the vertical length and transverse length of eggs were measured by a vernier caliper. The egg shape index was calculated by the ratio of the vertical length of the egg to the transverse length of the egg. Eggshell thickness and eggshell strength were measured using Egg shell thickness Gauge and Egg Force Reader (ORKA Food Technology Ltd, Ramat Hasharon, Israel). The albumen height, yolk color and HU were measured by Egg Analyzer (ORKA Food Technology Ltd, Israel). The concentrations of plasma immunoglobulin A (IgA, kit No H108), IgM (kit No H109), and IgG (kit No H106) were determined by automatic biochemical analyzer (Hitachi 7020; Hitachi High Technologies, Inc., Ibaraki, Japan). The above kits were provided by Nanjing Jiangcheng Biotechnology Co., Ltd (Nanjing, China). The activities of serum malondialdehyde (MDA, kit No A003-1-2), glutathione peroxidase (GSH-Px, kit No A005-1-2), superoxide dismutase (SOD, kit No A001-1-2), and catalase (CAT, kit No A007-1-1) were determined by using respective assay kits (Nanjing Jiangcheng Biotechnology Co., Ltd, China).

### Statistical analysis

The experimental data were analyzed by the general linear model procedure of SAS software (SAS Institute, 2003). When dietary therapy was significant (p<0.05), the mean values were compared by Duncan’s multiple comparison program (SAS Institute, 2003) of SAS software. The probability level of p< 0.05 was considered to be statistically significant.

## RESULTS

### Laying production performance

As shown in Table 2, dietary PR supplementation tended to increase daily feed intake (p = 0.0512). In addition, hens’ egg production tended to increase with the increasing of dietary PR concentrations (p = 0.0943), and the highest egg production was seen in the 1,200 mg/kg astaxanthin rich PR group. However, no significant differences were observed on average egg weight and feed conversion ratio among groups in the whole experimental period (p>0.05).

### Egg quality

There was no effect of astaxanthin rich PR on Haugh units, albumen height, egg shape index, eggshell strength, and eggshell thickness at weeks 6 (Table 3, p>0.05). In our study, adding astaxanthin rich PR to the layer diet significantly enhanced the yolk color (p<0.05). Egg shell weight tended to increase with the increasing of dietary PR concentrations (p = 0.0511).

### Antioxidant defenses

The results showed that the SOD and CAT were significantly increased in 800 and 1,200 mg/kg astaxanthin rich PR treatments compared with the control treatment (p<0.05). However, the MDA concentration significantly decreased in 800 and 1,200 g/kg astaxanthin rich PR treatments compared with the control treatment (p<0.05), and the suitable dosage was 1,200 mg/kg (Table 4).

### Immune defenses

Table 5 shows the content of serum immunoglobulin in experimental hens. The results showed that the content of IgG in serum was significantly increased in groups 800 and 1,200 mg/kg astaxanthin rich PR (p<0.05), which indicated that the appropriate amount of astaxanthin could improve the immunity of laying hens, and the optimal amount of astaxanthin was 1,200 mg/kg.

## DISCUSSION

In general, a traditional carotenoid source in poultry diets is β-carotene [17,18]. There is also a gradual trend to use *Haematococcus* astaxanthin to produce carotenoid-enriched eggs [6,19]. However, there is little research on the effect of astaxanthin rich PR in diets on laying hens, and our study was undertaken to help rectify this deficiency. In fact, the increase of daily egg production may relate to the fact that more feed was consumed with the increasing astaxanthin rich PR levels [20]. To date, the effect of carotenoid-enriched foods in poultry diets on performance of poultry is always a subject of debate. For example, feed intake has been reported to remain unchanged [21] or increase [22] upon astaxanthin-enriched foods supplementation. This discrepancy may be attributed to differences in diets composition and nutrition levels, and hen×s age and strain among the experiments.

Adding carotenoids to poultry diets can increase the oxidative stability of poultry products [23]. However, this study shows that there was no effect of astaxanthin rich PR on Haugh units (HU), albumen height, egg shape index, eggshell strength, and eggshell thickness at week 6. Egg yolk is the main carrier of egg flavor substances, and it is also an important index for consumers to evaluate the quality of eggs. The yolk color is an important index of egg quality. In our study, adding astaxanthin rich PR to the layer diet significantly enhanced the yolk color. A similar phenomenon was also found in other carotenoid studies [24]. Walker et al [19] studied the effects of palm toco and algae astaxanthin on egg quality parameters, and they found yolk color index was higher in algae astaxanthin group compared to palm toco group. Meanwhile, yolk color was changed (more red) by adding astaxanthin to laying hens’ diets [6,25]. Generally, carotenoids are the main pigments in animals [26]. Astaxanthin can be directly stored in tissues without modification or biochemical transformation after being absorbed by animals [27], which makes the skin and eggs of some animals appear healthy golden yellow or red. However, the content of polyunsaturated fatty acid in yolk could affect the yolk color and it could be improved by the addition of microalgal astaxanthin to the diet of laying hens [6,28].

Our research shows that astaxanthin red fermentation could significantly increase the activity of SOD and CAT and decrease the content of MDA in the serum of laying hens. Astaxanthin is a kind of carotene, one of the strongest natural antioxidants, which can effectively eliminate oxygen free radicals in LS-180 cells [29]. The molecular structure of astaxanthin is composed of many conjugated double bonds, and α-hydroxyketone is composed of ketone group and hydroxyl group at the end of the conjugated double bond chain [30]. These molecular structure characteristics of astaxanthin determine that it has an active electronic effect, which can provide electrons to free radicals or attract unpaired electrons of free radicals, so it can play the role of scavenging free radicals and antioxidation [31].

This experimental study shows that the content of IgG in serum was significantly increased, which indicated that the appropriate amount of astaxanthin could improve the immunity of laying hens. *In vitro* experiments showed that astaxanthin can improve the activity of T cells and the ability of peripheral blood monocytes to produce immunoglobulin [32]. In addition, it was found that the addition of astaxanthin rich PR to broiler diets had a positive effect on T cell proliferation and serum IgG content [33]. So, our results showed the increase in serum IgG content might be related to T cell proliferation. On the other hand, astaxanthin can protect the integrity of immune cells and ensure the normal immune response process [34], or it can improve the content of immunoglobulin by promoting the secretion of cytokines [35]. The mechanism of astaxanthin improving immune function may also be that it can promote the feeding and absorption of nutrients and provide necessary nutrients for the production of immune protein. This study shows that astaxanthin has no obvious effect on serum IgA and IgM levels, and its mechanism is not clear, which needs further study.

In summary, dietary astaxanthin rich PR supplementation had a remarkable influence on hens laying performance and egg quality, especially the improvement of yolk color. However, dietary astaxanthin rich PR supplementation in laying hens improved antioxidant defenses, and increased serum IgG contents. It suggested that astaxanthin rich PR can be utilized as a feed additive to improve the immunity and antioxidant capacity of laying hens.

## Figures and Tables

**Table 1:** Ingredients and diet composition (as-fed basis)

Item	Control	Astaxanthin rich *Phaffia rhodozyma* supplemental levels (mg/kg)		

		800	1,200	1,600
Ingredient (%)				
Corn	63.00	62.92	62.88	62.84
Soybean meal (48% CP)	20.00	20.00	20.00	20.00
Limestone	9.00	9.00	9.00	9.00
Wheat bran	3.00	3.00	3.00	3.00
*Phaffia rhodozyma*	0	0.08	0.12	0.16
Premix1)	5.00	5.00	5.00	5.00
Total	100	100	100	100
Composition (%)				
ME2) (MJ/kg)	10.98	10.98	10.98	10.98
Crud protein	15.30	15.30	15.31	15.31
Calcium	3.49	3.49	3.49	3.49
Available phosphorus	0.36	0.36	0.36	0.36
Methionine	0.72	0.72	0.72	0.72
Lysine	0.34	0.34	0.34	0.34
Methionine+cysteine	0.59	0.59	0.59	0.59
Tryptophan	0.18	0.18	0.18	0.18

1) Provided per kilogram of diet: 11,000 IU vitamin A; 3,700 IU vitamin D_3_; 35 mg vitamin E; 2.45 mg vitamin K_3_; 4 mg vitamin B_1_; 5 mg riboflavin; 4 mg vitamin B_6_; 4 mg vitamin B_12_; 60 mg niacin; 2.5 mg folic acid; 0.5 mg biotin; 20 mg pantothenic acid; 90 mg Zn; 105 mg Mn; 100 mg Fe; 8 mg Cu; 0.88 mg I; and 0.38 mg Se.

2) Metabolizable energy was calculated based on diet composition.

**Table 2:** Effect of dietary astaxanthin rich *Phaffia rhodozyma* levels on production performance of laying hens after 6 weeks of feeding

Items	Control	*Phaffia rhodozyma* supplemental levels (mg/kg)			p-value

		800	1,200	16,00	
Average egg weight (g)	64.51±1.781	63.84±1.89	63.31±2.11	63.70±2.54	0.5245
Egg production (%)	83.93±3.34	87.35±3.62	90.85±2.97	86.95±3.13	0.0943
Daily feed intake (g/bird)	122.04±3.06	123.65±3.08	126.07±2.96	126.02±3.11	0.0512
Feed conversion ratio	2.26±0.11	2.15±0.1	2.20±0.09	2.225±0.09	0.6567

**Table 3:** Effect of dietary astaxanthin rich *Phaffia rhodozyma* levels on egg quality of laying hens

Items	Control	*Phaffia rhodozyma* supplemental levels (mg/kg)			p-value

		800	1,200	1,600	
Egg shell weight (g)	6.69±0.54	7.02±0.55	6.9±0.59	6.99±0.53	0.0511
Egg-Shaped index	1.30±0.04	1.32±0.04	1.32±0.04	1.33±0.04	0.7356
Eggshell Strength (kg/cm^2^)	4.13±0.26	4.26±0.23	4.23±0.25	4.21±0.21	0.2333
Eggshell thickness (mm)	0.32±0.02	0.33±0.02	0.33±0.02	0.32±0.02	0.4345
Albumen height (mm)	6.80±0.36	6.94±0.38	6.99±0.39	6.94±0.38	0.6452
Yolk color	6.67±0.48^c^	7.48±0.43^b^	7.75±0.45^ab^	7.93±0.46^a^	0.0021
Haugh unit	80.15±4.26	82.63±3.55	82.52±3.8	80.27±3.21	0.2341

a–c Means in a row without a common superscripts differ (p<0.05).

**Table 4:** Effect of dietary astaxanthin rich *Phaffia rhodozyma* levels on antioxidant defenses of laying hens

Items	Control	*Phaffia rhodozyma* supplemental levels (mg/kg)			p-value

		800	1,200	1,600	
SOD (U/mL)	138.75±2.76^b^	158.75±3.26^a^	152.03±3.39^a^	145.36±3.25^ab^	0.0221
CAT (U/mL)	6.46±0.26^b^	6.86±0.29^a^	6.85±0.24^a^	6.71±0.33^ab^	0.0012
GSH-Px (μmol/L)	670.93±15.34	702.93±13.43	716.16±15.7	718.15±11.7	0.0538
MDA (nmol/mL)	2.90±0.41^a^	2.34±0.35^b^	2.26±0.38^b^	2.67±0.38^a^	0.0123

SOD, superoxide dismutase; CAT, catalase; GSH-Px, glutathione peroxidase; MDA, malondialdehyde.

a,b Means in a row without a common superscripts differ (p<0.05).

**Table 5:** Effect of dietary astaxanthin rich *Phaffia rhodozyma* levels on immune defenses of laying hens

Items	Control	*Phaffia rhodozyma* supplemental levels (mg/kg)			p-value

		800	1,200	1,600	
IgM (mg/L)	7.39±0.31	7.79±0.32	8.06±0.31	7.86±0.38	0.2278
IgA (mg/L)	19.06±0.65	20.01±0.64	20.51±0.65	20.11±0.64	0.1643
IgG (mg/L)	77.23±4.27^b^	86.21±3.92^a^	86.29±4.38^a^	84.34±4.05^ab^	0.0341

Ig, immunoglobulin.

a,b Means in a row without a common superscripts differ (p<0.05).
